# Prognostic value of oedema findings on head CT in traumatic brain injury following road traffic accidents

**DOI:** 10.3389/fradi.2026.1941750

**Published:** 2026-10-01

**Authors:** Diyou Chen, Wei Ma, Li Zhan, Hui Zhao, Kunlin Xiong

**Affiliations:** 1 Department of Radiology, Daping Hospital, Army Medical University, Chongqing, China; 2 Chongqing Clinical Research Centre of Imaging and Nuclear Medicine, Chongqing, China; 3 Chongqing Municipal Key Clinical Specialty (Excellence) Construction Platform, Chongqing, China; 4 Department of Wound Infection and Drug, State Key Laboratory of Trauma, Burn and Combined Injury, Daping Hospital, Army Medical University, Chongqing, China; 5 Institute for Traffic Medicine, Daping Hospital, Army Medical University, Chongqing, China

**Keywords:** cerebral oedema, ct score, functional prognosis, road traffic accidents, traumatic brain injury

## Abstract

**Introduction:**

Existing CT-based prognostic models for traumatic brain injury (TBI) are not tailored to specific injury mechanisms and lack sensitivity to cerebral oedema changes. This study aimed to develop an assessment method for cerebral oedema and compare it with conventional CT scoring systems in predicting long-term outcomes in patients with TBI following road traffic accidents (RTAs).

**Methods:**

A retrospective analysis was conducted on 227 patients with TBI following RTAs admitted to a single center (2019.1-2023.12). A clinical prognostic model (IMPACT) was defined, comprising age, motor score, and pupillary light reflex. The A5 (+1) CT classification was defined based on the involvement of cerebral lobes by oedema and the resulting mass effect. The Marshall CT classification, Rotterdam CT score, Helsinki CT score, and A5 (+1) CT classification were added to the IMPACT model to assess their value in predicting outcomes using area under the curve (AUC).

**Results:**

The AUC for A5 (+1) CT classification reached 0.849, surpassing Marshall CT classification (0.720, *p* < 0.001), Rotterdam CT score (0.745, *p* = 0.004), and Helsinki CT score (0.746, *p* = 0.002). Adding A5 (+1) CT classification to IMPACT model increased the AUC by 0.076 (*p* = 0.001). Adding Rotterdam CT score increased the AUC by 0.038 (*p* = 0.028), while incorporating Marshall CT classification and Helsinki CT score yielded no significant changes (*p* = 0.138, *p* = 0.057).

**Discussion:**

The A5 (+1) CT classification outperforms conventional CT scoring systems in assessing long-term outcomes for patients with TBI following RTAs. Its inclusion in the IMPACT model enhances predictive performance.

## Introduction

Globally, traumatic brain injury (TBI) represents a significant public health threat to human wellbeing (1–3). Conventional management encompasses monitoring hypotension, hypoxia, and intracranial pressure, alongside surgical intervention (4). Reliable prediction of functional outcomes facilitates clinical decision-making and informed therapeutic strategies (5). Currently, the International Mission for Prognosis and Analysis of Clinical Trials in TBI (IMPACT) represents the most mature predictive model for unfavorable functional outcomes at 6 months (6). A growing body of research indicates that early structural abnormalities detected by computed tomography (CT) may possess comparable predictive value (7–9).

The Marshall CT classification, Rotterdam CT score, and Helsinki CT score are widely employed CT-based prognostic scoring systems for TBI (10–12). These have evolved from qualitative to quantitative assessments, shifting focus from morphology to volume, thereby enhancing their predictive efficacy (12). However, most such scoring systems rely on non-contrast CT (NCCT) at a single time point (typically the initial post-admission scan), failing to capture progressive pathological processes such as secondary injury. Cerebral oedema constitutes a key pathophysiological manifestation of TBI and serves as a marker of secondary injury (13, 14). The value of detecting cerebral oedema on NCCT in TBI prognosis assessment remains unclear.

Road traffic accidents (RTAs) represent the primary cause of TBI (15) and rank as the leading cause of injury in low- and middle-income countries (16). TBI following RTAs exhibits unique biomechanical features, typically arising from acceleration-deceleration and rotational shear forces (17). As a result, these injuries carry a markedly higher risk of diffuse axonal injury and widespread vasogenic oedema relative to other trauma mechanisms such as falls (18). Currently, TBI prognostic models are applicable to patient cohorts with diverse injury etiologies, whilst studies examining their predictive value for TBI patients with a single injury cause remain scarce. Although conventional CT scoring systems can detect midline shift and basal cistern compression, their scoring weights are predominantly determined by hematoma volume or ventricular dimensions. Consequently, they fail to capture the spatial distribution of cerebral oedema across different injury mechanisms or the progressive changes it induces (19). This limitation may lead to underestimation of the delayed impact of oedema on the brainstem and critical functional regions within this particular RTAs subgroup. This study exclusively included patients with TBI following RTAs to evaluate the predictive value of the IMPACT model, CT scoring systems, and cerebral oedema parameters for long-term functional outcomes. We hypothesize that incorporating cerebral oedema parameters into the IMPACT model's outcome predictors may enhance its predictive performance.

## Methods

### Patients

A retrospective cohort study was conducted between January 2019 and December 2023 at a trauma center in Chongqing, China, involving 227 adult patients with TBI resulting from RTAs. Approval was obtained from the Ethics Committee of Daping Hospital, Army Medical University (Approval No. 2023194). Our study was conducted in accordance with the Declaration of Helsinki.

Patients were only included if admitted with acute traumatic intracerebral abnormalities on initial NCCT. As surgical procedures such as decompressive craniectomy or hematoma evacuation may induce hemodynamic alterations affecting cerebral oedema progression, patients undergoing such operations within four days post-injury were excluded from this study. Monitoring procedures such as intracranial pressure probe placement and external ventricular drainage were not excluded. The patient flow diagram is presented in Figure 1.

**Figure 1:**
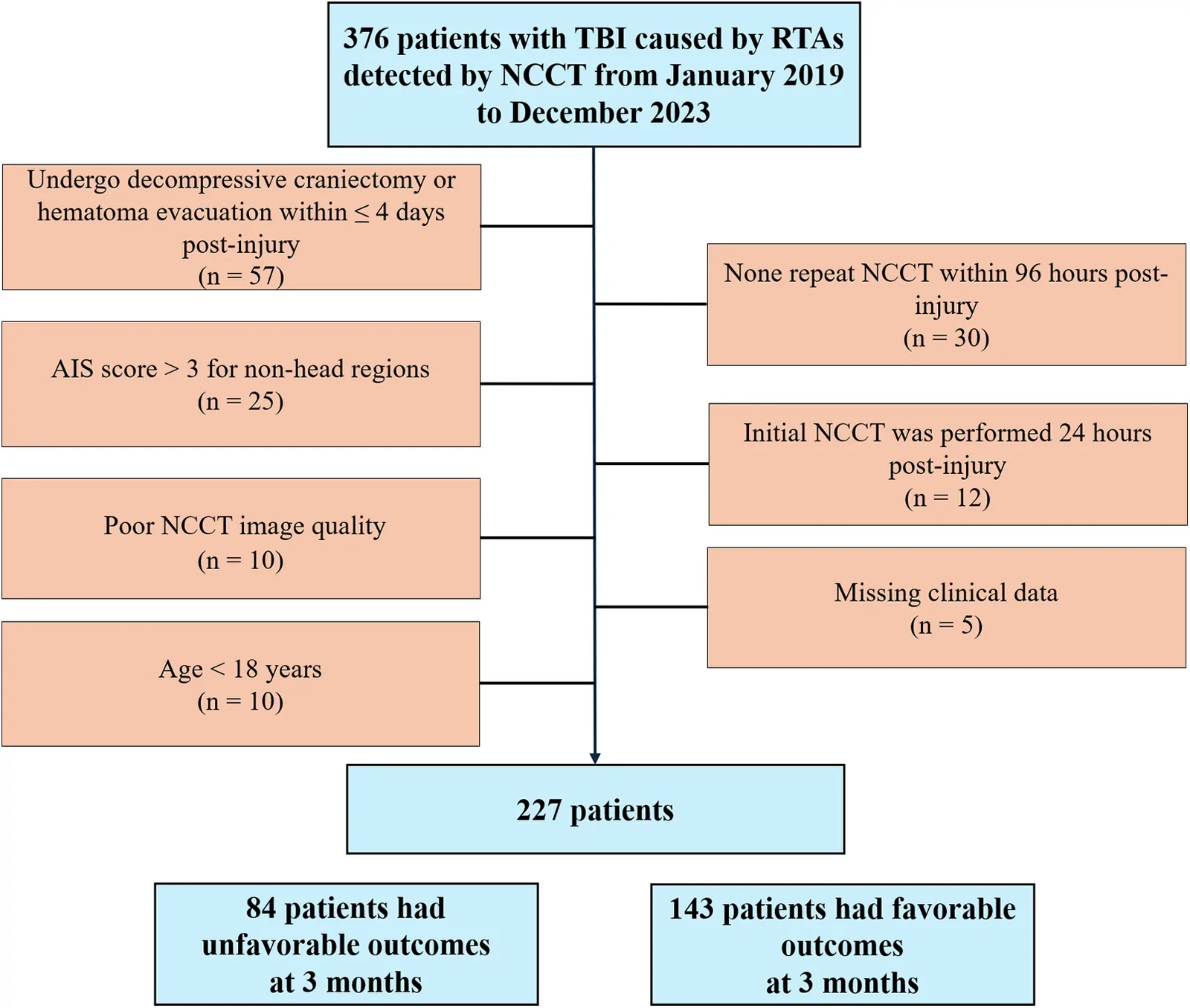
Flowchart of the study. In total, 227 of 376 patients from one trauma center were enrolled in this study.

## Imaging protocol and data analysis

Given that cerebral oedema typically peaks between 2 and 4 days post-injury (20), prognostic evaluation based on early data is crucial for supporting initial clinical decision-making. Patients enrolled in this study underwent head NCCT using either a GE 64-slice vCT or a Philips 128-slice iCT scanner at least once within each of two timeframes: within 24 h post-injury and between 2 and 4 days post-injury. The scanning range extends from the cranial vault to the foramen magnum, comprising 24 slices. Specific parameters: GE 64-slice vCT: Slice thickness: 5 mm, Slice spacing: 5 mm, Tube voltage: 120 kV, Tube current: 350 mA. Philips 128-slice iCT: Slice thickness: 5 mm, Slice spacing: 5 mm, Tube voltage: 120 kV, Tube current: 400 mA.

The Marshall CT classification, Rotterdam CT score, and Helsinki CT score were determined from the initial NCCT findings. Concurrently, the presence of skull fractures, subdural hematoma, epidural hematoma, intraventricular hemorrhage, intracerebral hemorrhage [ICH]/contusion (locations and numbers), midline shift (> 5 mm), and cerebral oedema were documented.

Utilizing the most pronounced cerebral oedema observed on NCCT within 96 h post-injury, the volume of ICH/contusion was calculated using the Tada formula. This method has demonstrated accuracy in measuring ICH and inter-observer reliability (12). The volume of perilesional oedema surrounding ICH/contusion lesions was calculated using 3D Slicer software (version 5.0, CSAIL) (21). The oedema index (EI) was introduced to assess the severity of oedema in patients with concomitant ICH/contusion-associated cerebral oedema, as detailed in Equation 1 (Figure 2). The A5 (+1) CT classification represents an alternative method for evaluating oedema severity, focusing on its distribution across cerebral lobes within the cranium and the resulting mass effect (22, 23) (Table 1; Figure 3).

$$\mathrm{EI}=\left(V_{\mathrm{edema}}+V_{\mathrm{contusion}/\mathrm{ICH}}\right)/V_{\mathrm{contusion}/\mathrm{ICH}}$$ (1)

**Figure 2:**
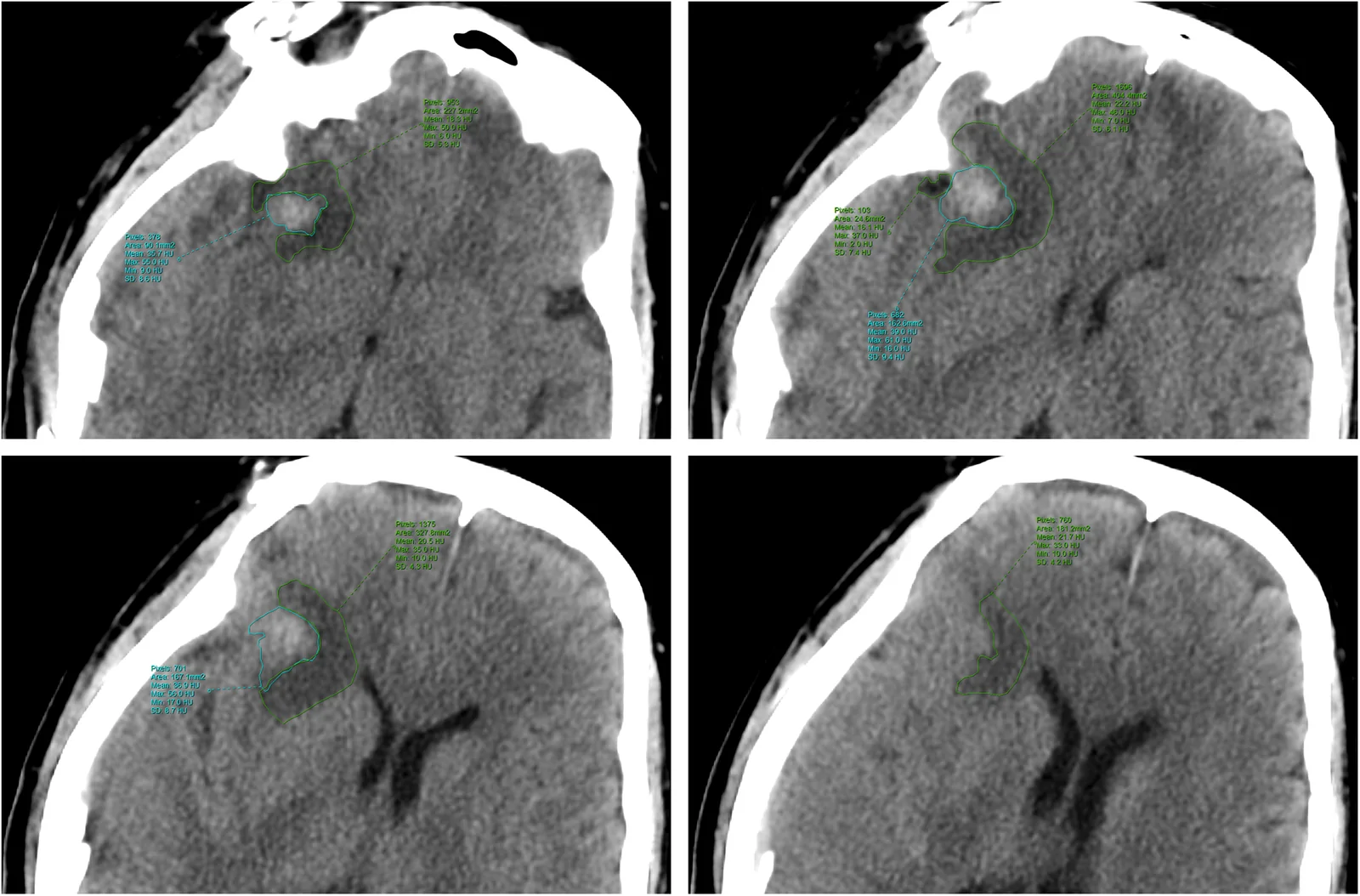
Diagram of the hematoma and oedema.

**Table 1:** The standard of A5 (+1) CT classification.

Grade	NCCT imaging findings
0	No oedema
1	Oedema confined to a single cerebral lobe
2	Multiple cerebral lobes with oedema, confined to a single hemisphere
3	Bilateral cerebral oedema
4	Disappearance of sulci, with visible diffuse oedema in the basal cisterns
5	Diffuse oedema with disappearance of both sulci and basal cisterns
+1	Additional point: Oedema causing mass effect resulting in midline shift > 5 mm (for grades 1–4)

**Figure 3:**
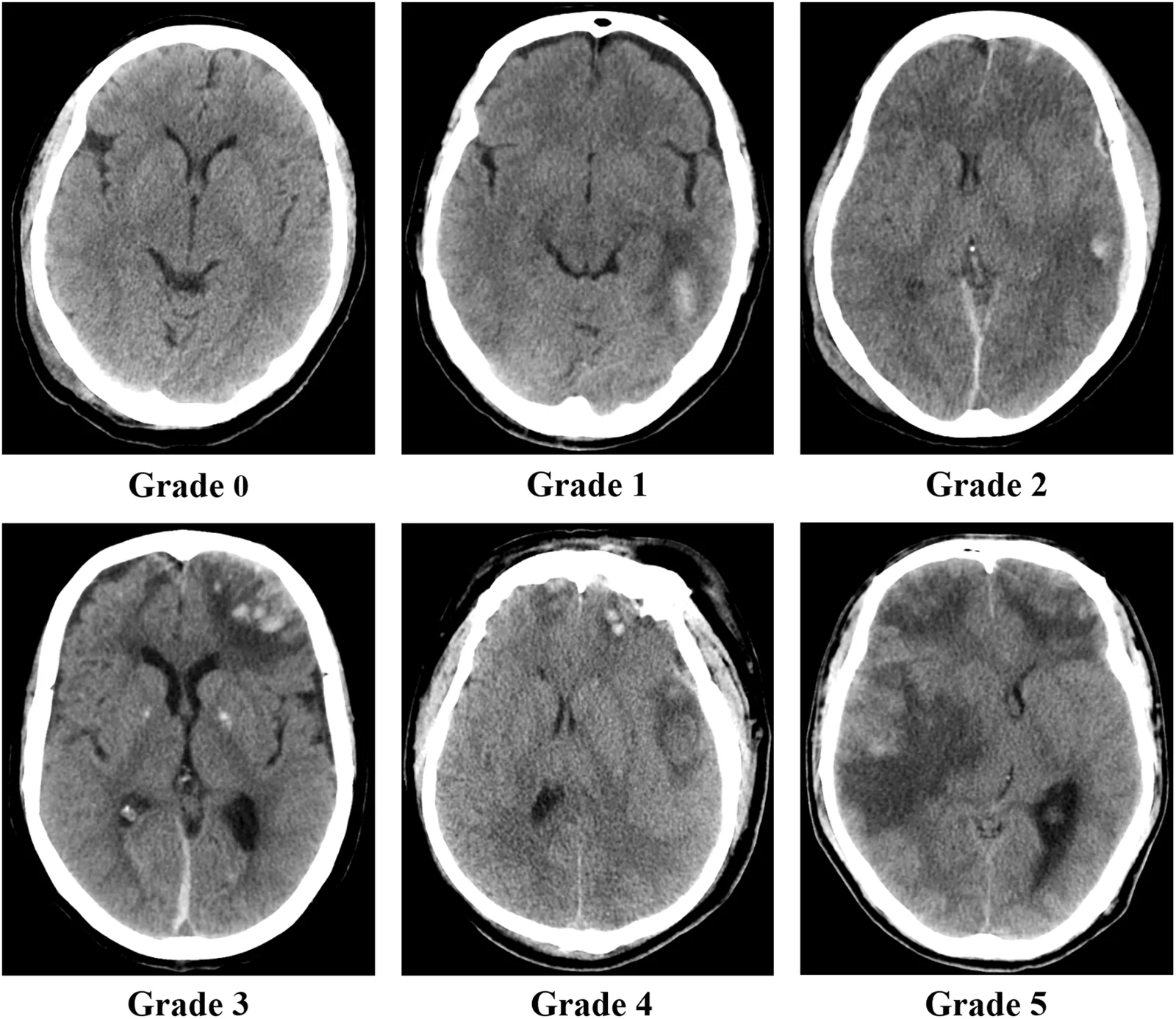
Reproduced with permission from Ref 23. with > Reproduced from “Appendix A. FIG. S3. Schematic diagram of A5 (+1) CT classification” by D. Chena, L. Zhana, X. Shic, X. Zhub, J. Xieb, J. Qiub, Q. Xiac, H. Zhaob and K. Xiong, licensed under CC BY-NC-ND.

Where, EI denotes the oedema index, V denotes volume, and ICH denotes intracerebral hemorrhage.

### Clinical data

Patient demographics, roles in RTAs, pupillary light reflex, head AIS score, number of intracranial lesion sites. Additionally, motor score, Glasgow Coma Scale (GCS), time to initial NCCT scan, and length of hospital stay were recorded. TBI severity was defined according to GCS (24): mild TBI (13–15 points), moderate TBI (9–12 points), and severe TBI (3–8 points). Structured follow-up was conducted three months post-injury, with functional outcomes categorized as favorable [Glasgow Outcome Scale (GOS) score > 3] or unfavorable (GOS score ≤ 3) using the GOS (25).

### Prognosis models

The IMPACT model comprises age at admission, motor score, and pupillary light reflex (26). Additionally, the Marshall CT classification, Rotterdam CT score, Helsinki CT score, and A5 (+1) CT classification are incorporated. Additionally, we incorporated Marshall CT classification, Rotterdam CT score, Helsinki CT score, and A5 (+1) CT classification into the IMPACT model for combined prognostic assessment. Finally, we conducted a subgroup analysis for patients with cerebral oedema and added EI to the IMPACT model.

### Statistical analysis

This study adopted a multi-software coordinated statistical analysis strategy to perform consistency checks for overlapping primary analytical results. General statistical analysis was conducted using SPSS (version 25.0, IBM). Patients were categorized based on 3-month GOS. Continuous variables not meeting normality were expressed as median [interquartile range (IQR)] and compared between groups using the Mann–Whitney U test. Categorical data were presented as *n* (%), with intergroup comparisons performed using chi-square tests or R*C contingency table analyses. A *p*-value < 0.05 was considered statistically significant.

Receiver operating characteristic (ROC) curves were plotted using MedCalc (version 2021, MedCalc Soft Lt) to analyze and evaluate each prognostic model, incorporating area under the curve (AUC). The DeLong test was employed to compare differences in AUC between various models. To further validate the clinical utility of oedema parameters, the R Project for Statistical Computing (version 4.5.0, GNU project) was employed to report the net reclassification index (NRI), integrated discrimination improvement (IDI), and decision curve analysis (DCA) results.

## Results

### Participants

A total of 376 patients with TBI following RTAs met the inclusion criteria. Of these, 149 were excluded: 57 underwent decompressive craniectomy or hematoma evacuation within ≤ 4 days post-injury, 30 had no repeat NCCT within 96 h, 25 had an AIS score > 3 for non-head regions, 12 had an initial NCCT performed 24 h post-injury, 10 had poor NCCT image quality, 10 were aged < 18 years, and 5 had missing clinical data. The final study cohort consisted of 227 patients. Baseline characteristics are presented in Table 2.

**Table 2:** Baseline characteristics of 227 patients enrolled in the study.

Variable	All patients
Male, *n* (%)	166 (73.1)
Age, median (IQR)	48 (22)
Road user roles, *n* (%)	
Motor vehicle driver	21 (9.3)
Passenger in motor vehicle	21 (9.3)
Motorcyclist	95 (41.8)
Pedestrian	90 (39.6)
GCS, *n* (%)	
3–8	82 (36.1)
9–12	47 (20.7)
13–15	98 (43.2)
Motor score, *n* (%)	
1	14 (6.2)
2	33 (14.5)
3	73 (32.1)
4	46 (20.3)
5–6	61 (26.9)
Pupillary light reflex, *n* (%)	
Normal	167 (73.6)
Unilateral disappearance	27 (11.9)
Bilateral disappearance	33 (14.5)
Head AIS, median (IQR)	3 (2)
Multiple intracranial lesions, *n* (%)	150 (66.1)
Length of hospital stay, median (IQR)	18 (18)
Initial NCCT time, *n* (%)	
< 6 h	197 (86.8)
≥ 6 h and < 12 h	14 (6.1)
≥ 12 h and < 24 h	16 (7.1)
GOS, *n* (%)	
1	34 (14.9)
2	31 (13.7)
3	19 (8.4)
4	72 (31.7)
5	71 (31.3)

IQR, interquartile range; GOS, Glasgow Coma Scale; AIS, Abbreviated Injury Scale; NCCT, non-contrast CT; GOS, Glasgow Outcome Scale.

### Comparison of clinical data between patients with different functional outcomes at 3 months

Grouped by functional outcome at 3 months, univariate analysis revealed that patients with favorable outcomes were on average 6 years younger than those with unfavorable outcomes (*p* = 0.011) and had lower head AIS scores (3 vs. 4, *p* < 0.001). Significant differences were also observed in motor score, GCS, and pupillary light reflex (all *p* < 0.001), whereas no differences were noted in gender or road user roles (*p* = 0.17, 0.056) (Supplementary Table S1).

### Comparison of imaging data for different functional outcomes at 3 months

Ventricle hemorrhage, subarachnoid hemorrhage, midline shift, and cerebral oedema were more prevalent in patients with unfavorable outcomes (*p* < 0.001). Furthermore, those with unfavorable outcomes exhibited a higher number of contusions and greater involvement of cerebral lobes (*p* = 0.01, *p* < 0.001), along with higher proportions of Rotterdam CT scores, Helsinki CT scores, and Marshall CT classification grades IV and above (all *p* < 0.001) (Supplementary Table S2).

Further analysis of patients exhibiting cerebral oedema on NCCT revealed that those with unfavorable outcomes demonstrated significantly higher oedema volume (5.463 mL vs. 1.343 mL, *p* < 0.001) and EI (2.864 vs. 2.287, *p* < 0.001). The A5 (+1) CT classification predominantly fell within grades 3–5 (71.2%), whereas those with favorable outcomes were mainly classified as grades 1–2 (77.6%) (*p* < 0.001) (Supplementary Table S3).

### The predictive performance of prognostic models for 3-month functional outcomes in patients with TBI following RTAs

ROC analysis across all enrolled patients yielded an AUC of 0.838 (95% CI: 0.783, 0.883) for the IMPACT model (Table 3), surpassing Marshall CT classification (*p* = 0.003), Rotterdam CT score (*p* = 0.019), and Helsinki CT score (*p* = 0.026). The AUC for A5 (+1) CT classification reached 0.849 (95% CI: 0.795, 0.893), surpassing Marshall CT classification (*p* < 0.001), Rotterdam CT score (*p* = 0.004), and Helsinki CT score (*p* = 0.002) (Figures 4A, 5A).

**Table 3:** Predictive value of different prognosis models (all included patients).

Variable	AUC (95% CI)	*p* value
IMPACT	0.838 (0.783–0.883)	< 0.001
Marshall CT classification	0.720 (0.653–0.788)	< 0.001
Rotterdam CT score	0.745 (0.677–0.813)	< 0.001
Helsinki CT score	0.746 (0.678–0.813)	< 0.001
A5 (+1) CT classification	0.849 (0.795–0.893)	< 0.001
IMPACT + A5 (+1) CT classification	0.914 (0.869–0.947)	< 0.001
IMPACT + Marshall CT classification	0.855 (0.802–0.898)	< 0.001
IMPACT + Rotterdam CT score	0.876 (0.826–0.916)	< 0.001
IMPACT + Helsinki CT score	0.867 (0.819–0.909)	< 0.001

IMPACT, International Mission for Prognosis and Analysis of Clinical Trials in TBI.

**Figure 4:**
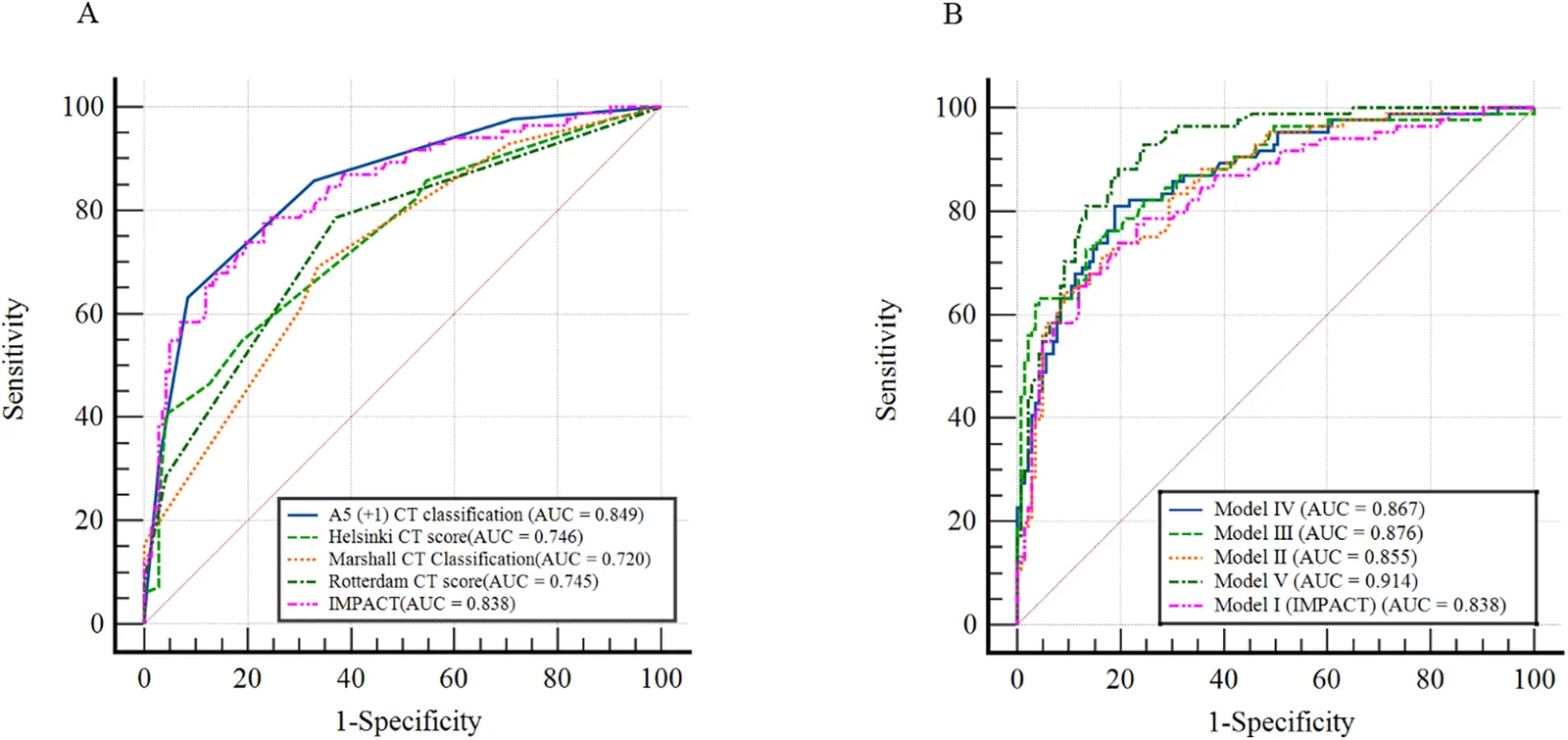
Receiver operating characteristic curves of the prognosis models (all included patients). **(A)** Single model (*n* = 227). **(B)** Joint model (*n* = 227). Model Ⅰ (IMPACT): Age + Pupillary light reactivity + Motor score; Model Ⅱ: IMPACT + Marshall CT classification; Model Ⅲ: IMPACT + Rotterdam CT score; Model Ⅳ: IMPACT + Helsinki CT score; Model Ⅴ: IMPACT + A5 (+1) CT classification. IMPACT, International Mission for Prognosis and Analysis of Clinical Trials in TBI.

**Figure 5:**
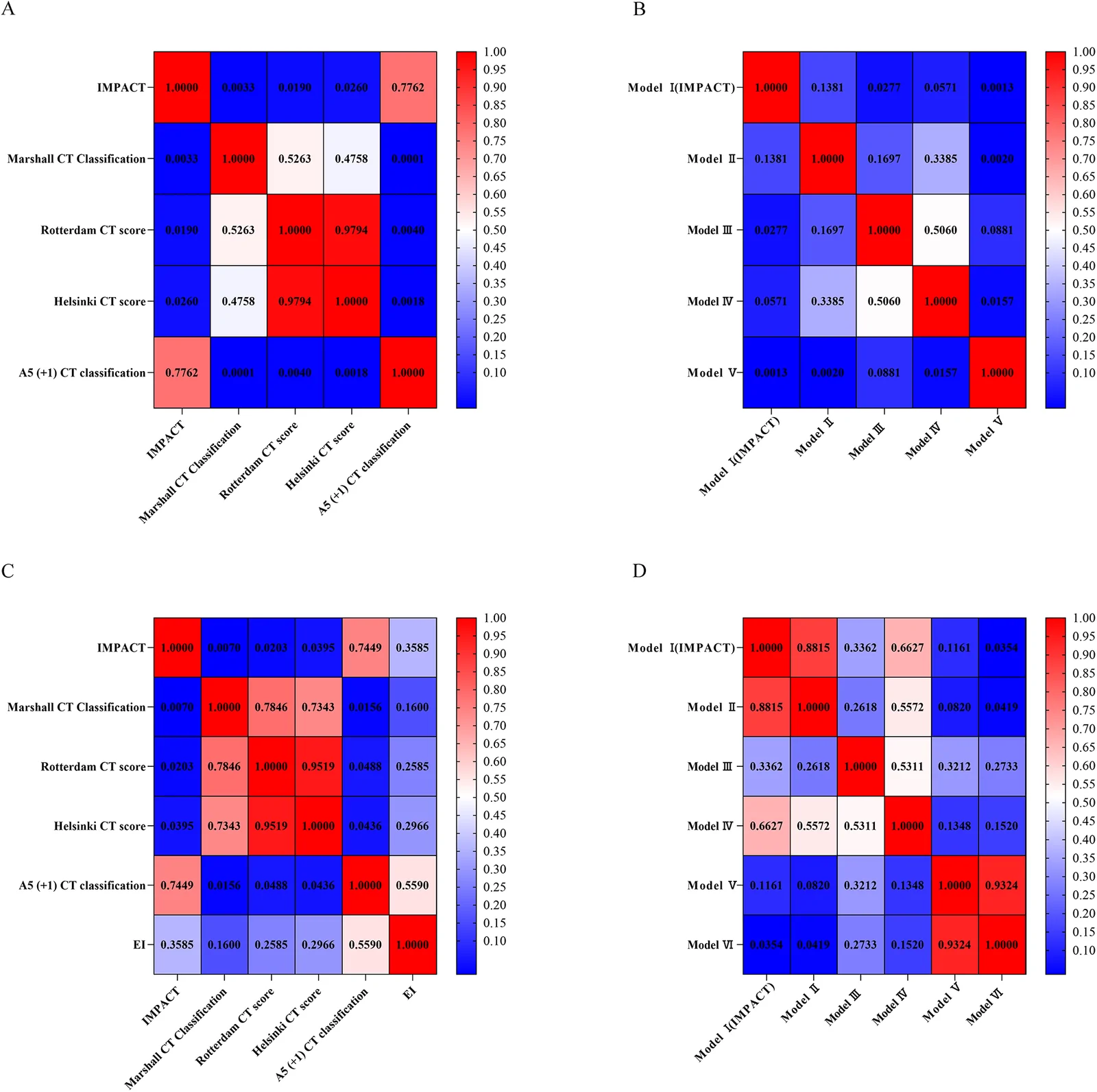
Heat maps of the prognosis models (all included patients). **(A,C)** Single model. **(B,D)** Joint model. **(A,B)** All included patients (*n* = 227). **(C,D)** Patients with concurrent cerebral oedema (*n* = 108). Model Ⅰ (IMPACT): Age + Pupillary light reactivity + Motor score; Model Ⅱ: IMPACT + Marshall CT classification; Model Ⅲ: IMPACT + Rotterdam CT score; Model Ⅳ: IMPACT + Helsinki CT score; Model Ⅴ: IMPACT + A5 (+1) CT classification; Model Ⅵ: IMPACT + EI. IMPACT, International Mission for Prognosis and Analysis of Clinical Trials in TBI; EI, oedema index.

When IMPACT model parameters were added to the Marshall CT classification, Rotterdam CT score, Helsinki CT score, and A5 (+1) CT classification respectively, all combined models demonstrated good discriminatory ability for adverse outcomes (AUC > 0.80) (Table 3). Compared to using the IMPACT model alone, the AUC increased by 0.0378 (*p* = 0.028) when combined with Rotterdam CT score, and by 0.0756 (*p* = 0.001) when combined with A5 (+1) CT classification (Figures 4B, 5B).

### The predictive performance of prognostic models for 3-month functional outcomes in patients with TBI following RTAs with concurrent cerebral oedema

For patients with concomitant cerebral oedema, the AUC obtained by IMPACT model was 0.798 (95% CI: 0.710, 0.869), surpassing Marshall CT classification (*p* = 0.007) (Table 4), Rotterdam CT score (*p* = 0.02), and Helsinki CT score (*p* = 0.039). The AUC for A5 (+1) CT classification reached 0.778 (95% CI: 0.688, 0.853), surpassing Marshall CT classification (*p* = 0.016), Rotterdam CT score (*p* = 0.049), and Helsinki CT score (*p* = 0.044) (Figures 5C, 6A).

**Table 4:** Predictive value of different prognosis models (patients with concurrent cerebral oedema).

Variable	AUC (95% CI)	*p* value
IMPACT	0.798 (0.710–0.869)	< 0.001
Marshall CT classification	0.646 (0.543–0.749)	0.009
Rotterdam CT score	0.661 (0.560–0.763)	0.004
Helsinki CT score	0.664 (0.561–0.767)	0.003
A5 (+1) CT classification	0.778 (0.688–0.853)	< 0.001
EI	0.740 (0.647–0.820)	< 0.001
IMPACT + A5 (+1) CT classification	0.851 (0.770–0.912)	< 0.001
IMPACT + Marshall CT classification	0.796 (0.708–0.868)	< 0.001
IMPACT + Rotterdam CT score	0.817 (0.731–0.885)	< 0.001
IMPACT + Helsinki CT score	0.806 (0.718–0.875)	< 0.001
IMPACT + EI	0.848 (0.766–0.910)	< 0.001

IMPACT, International Mission for Prognosis and Analysis of Clinical Trials in TBI; EI, oedema index.

**Figure 6:**
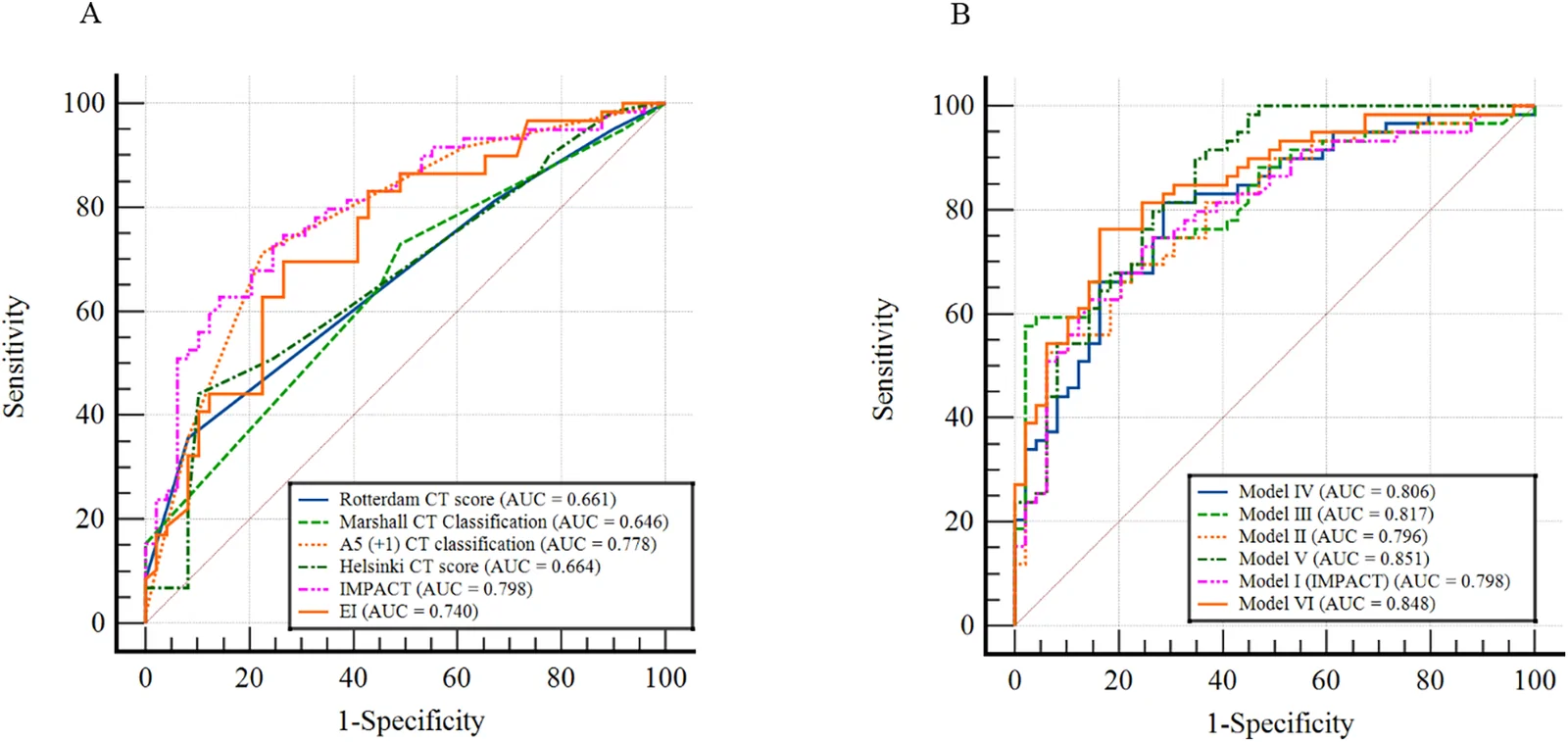
Receiver operating characteristic curves of the prognosis models (patients with concurrent cerebral oedema). **(A)** Single model (*n* = 108). **(B)** Joint model (*n* = 108). Model Ⅰ (IMPACT): Age + Pupillary light reactivity + Motor score; Model Ⅱ: IMPACT + Marshall CT classification; Model Ⅲ: IMPACT + Rotterdam CT score; Model Ⅳ: IMPACT + Helsinki CT score; Model Ⅴ: IMPACT + A5 (+1) CT classification; Model Ⅵ: IMPACT + EI. IMPACT, International Mission for Prognosis and Analysis of Clinical Trials in TBI; EI, cerebral oedema.

After sequentially adding the Marshall CT classification, Rotterdam CT score, Helsinki CT score, A5 (+1) CT classification, and EI to the IMPACT model, compared with the IMPACT model alone, the combined AUC of IMPACT and EI increased by 0.05 (*p* = 0.035) (Table 4). However, the combined AUCs of the Marshall CT classification, Rotterdam CT score, Helsinki CT score, and A5 (+1) CT classification with the IMPACT model showed no significant differences (all *p* > 0.05) (Figures 5D, 6B).

### Analysis of the clinical utility of the prognosis model

Among all enrolled patients, the NRI value for adding A5 (+1) CT classification to the IMPACT model was 0.868 (0.613, 1.105), with an IDI of 0.168 (0.111, 0.219), indicating improved predictive capability (Supplementary Table S4). DCA (Figure 7A) demonstrated that this model conferred high-value benefits to patients within a high threshold probability range. In patients with concomitant cerebral oedema, the NRI value when incorporating EI into the IMPACT model was 0.670 (0.250, 1.151), with an IDI of 0.107 (0.033, 0.243). Incorporating A5 (+1) CT classification into the IMPACT model yielded an NRI of 0.975 (0.540, 1.277) with an IDI of 0.134 (0.032, 0.286) (Supplementary Table S5). However, the DCA indicated that this model conferred high-value benefits to patients within a small scope (Figure 7B).

**Figure 7:**
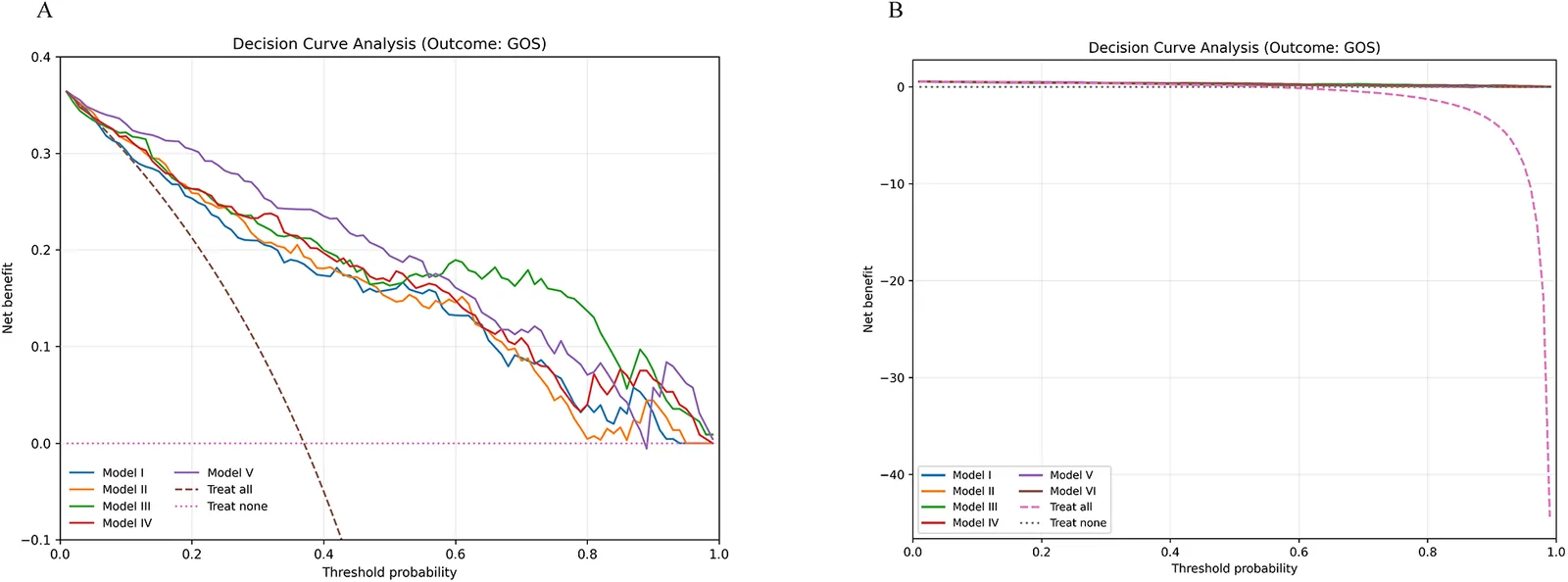
The clinical DCA of the joint model. **(A)** All included patients (*n* = 227). **(B)** Patients with concurrent cerebral oedema (*n* = 108). Model Ⅰ (IMPACT): Age + Pupillary light reactivity + Motor score; Model Ⅱ: IMPACT + Marshall CT classification; Model Ⅲ: IMPACT + Rotterdam CT score; Model Ⅳ: IMPACT + Helsinki CT score; Model Ⅴ: IMPACT + A5 (+1) CT classification; Model Ⅵ: IMPACT + EI. IMPACT, International Mission for Prognosis and Analysis of Clinical Trials in TBI; EI, cerebral oedema.

## Discussion

We conducted a single-center retrospective study to investigate the predictive value of the IMPACT model, Marshall CT classification, Rotterdam CT score, Helsinki CT score, and cerebral oedema parameters for three-month functional outcomes in patients with TBI following RTAs. Cerebral oedema parameters demonstrated superior efficacy. Incorporating cerebral oedema parameters into the established outcome predictors of the IMPACT model yielded markedly improved predictive performance.

Previous studies have rarely explored the prognostic value of CT scoring systems for patients with TBI resulting from a single cause of injury. This study focused on RTAs, the primary cause of TBI in low- and middle-income countries (2). This aligns with previous reports indicating that CT scoring systems based on NCCT demonstrate limited prognostic value for TBI resulting from multiple causes (10, 27). In this study, the predictive performance of the IMPACT model (AUC = 0.838) surpassed that of CT scoring systems. Incorporating the Marshall CT classification and Rotterdam CT score into the IMPACT model did not yield significant performance gains. This finding suggests that early NCCT features may hold limited predictive value for functional outcomes in patients with RTAs-induced TBI. A 2014 study demonstrated that incorporating the Helsinki CT score into the IMPACT model improved predictive performance for adverse neurological outcomes (AUC increase of 0.02) (12). In our study, incorporating the Helsinki CT score into the IMPACT model increased the AUC by 0.029, demonstrating a similar trend, though the difference was not statistically significant (*p* = 0.057). This outcome may be attributable to the relatively small sample size.

The IMPACT model (including IMPACT-core, IMPACT-extended, and IMPACT-lab) is recommended for predicting mortality from 14 days to 6 months after TBI, as well as functional outcomes at 6 months and beyond, with moderate predictive reliability. For ICU-admitted patients with moderate-to-severe TBI, the clinical prediction model combining IMPACT core with CT and laboratory parameters can serve as a moderately reliable tool for predicting 6-month mortality (28). Nevertheless, current reports are inconsistent. In one cohort of patients with moderate-to-severe TBI, the AUCs for mortality prediction using the Core model, CT model, and Laboratory model were 0.61, 0.771, and 0.822, respectively, while the AUCs for predicting 6-month poor outcomes were 0.601, 0.671, and 0.645. In another cohort, the AUCs of the three models for mortality prediction were 0.757, 0.802, and 0.794, respectively, and those for 6-month poor outcomes were 0.731, 0.698, and 0.605 (29). A separate validation study including 199 patients with moderate-to-severe TBI demonstrated that the IMPACT-lab model achieved excellent discriminative ability for poor outcomes (AUC = 0.887) and mortality (AUC = 0.880) (30). Our study focuses on the IMPACT core model combined with CT variables and does not explore the IMPACT-lab model.

Cerebral oedema is a prevalent pathological alteration in both TBI and ischemic stroke, yet substantial differences exist between the two conditions at multiple levels. Mechanistically, TBI is induced by mechanical forces that directly disrupt the blood–brain barrier, with subsequent inflammatory cascades further exacerbating vasogenic oedema. In contrast, the early stage of ischemic stroke is predominantly characterized by cytotoxic oedema resulting from energy metabolism failure (13, 31). In terms of temporal progression, traumatic cerebral oedema presents a biphasic pattern, and vasogenic oedema can persist for up to seven days after injury (32). By comparison, stroke-related oedema undergoes a sequential progression from cytotoxic oedema to vasogenic oedema, with the vasogenic component typically emerging 12 h after cerebral ischemia (33). With regard to spatial distribution, stroke-induced oedema is largely confined to the vascular territory of the ischemic lesion, whereas traumatic cerebral oedema manifests as more diffuse and heterogeneous lesions involving both local injured areas and remote brain regions (34). On this basis, the present study focuses on evaluating the severity and characteristics of cerebral oedema secondary to TBI following RTAs, aiming to more accurately clarify its prognostic value and provide targeted evidence for clinical decision-making in TBI management.

Cerebral oedema constitutes the main secondary injury following TBI and represents a common CT finding in RTAs-induced TBI (35). Currently, there is a lack of universally accepted and reliable assessment methods. Clinically, intracranial pressure monitoring serves as the principal method for evaluating cerebral oedema, despite its invasive and lagging nature (36). The Marshall CT classification (37) and the A5 (+1) CT classification (22, 38) have been successively reported for assessing cerebral oedema following TBI. Autopsy studies confirmed that brain water content in patients with Marshall CT Grade I and II TBI approached normal levels (37). Further investigations revealed median CT values of 14.31 HU and 22.48 HU in brain tissue from patients with Marshall CT classification grade III or higher versus grade I or II injuries (39), suggesting that grade III or higher oedema severity may be assessed as severe. The A5 (+1) CT classification, established based on the lobar distribution and mass effect of edematous lesions, effectively predicts functional outcomes in patients with cerebral contusions (17). Previously, we compared the Marshall CT classification and A5 (+1) CT classification for assessing the severity of contusion-related cerebral oedema and proposed a novel parameter (EI) combining ICH/contusion volume and oedema volume as an effective measure of cerebral oedema severity (23). In this study, 47.6% of enrolled patients presented with cerebral oedema. The A5 (+1) CT classification demonstrated superior predictive value for 3-month outcomes compared to the Marshall CT classification, Rotterdam CT score, and Helsinki CT score (*p* < 0.001, *p* = 0.004, *p* = 0.002, respectively). Incorporating the A5 (+1) CT classification into the IMPACT model increased the AUC by 0.076 (*p* = 0.001) and the NRI by 86.8%. This highlights the utility and necessity of adding cerebral oedema parameters to existing TBI prognostic models, particularly for RTAs-induced TBI.

We further conducted a separate analysis of patients with combined cerebral oedema and introduced the EI. We found that patients with unfavorable outcomes exhibited higher oedema volume and EI values compared to those with favorable outcomes. The A5 (+1) CT classification in patients with unfavorable outcomes was predominantly distributed across grades 3–5 (71.2%), whereas those with favorable outcomes were concentrated in grades 1–2 (77.6%). Concurrently, the A5 (+1) CT classification continued to demonstrate superior prognostic predictive performance compared to the Marshall CT grading, Rotterdam CT score, and Helsinki CT score. The AUC value of the combined EI and IMPACT model increased by 0.05 compared to the IMPACT model alone, and by 0.051 compared to the combined IMPACT and Marshall CT classification model. This may be attributed not only to the higher incidence of cerebral oedema in RTAs but also to the EI incorporating both ICH/contusion and oedema variables. Research confirms that quantitative measurement of cerebral contusion volume aids in predicting long-term functional outcomes (40), whilst cerebral oedema, as a significant pathophysiological manifestation of TBI, reflects its progressive nature (13, 14). The AUC for the A5 (+1) CT classification combined with the IMPACT model increased compared to the IMPACT model alone, though without significant difference. We propose the following reasons: firstly, during the acute phase of TBI with cerebral oedema, clinical interventions often prioritize dehydrating agents (41), leading to relatively increased density on NCCT and thereby obscuring radiographic oedema signs, potentially underestimating oedema severity. Secondly, the sample size for cases with cerebral oedema was relatively small.

In patients with concomitant cerebral oedema, incorporating the EI into the IMPACT model yielded an NRI of 0.670 and an IDI of 0.107. Following the addition of the A5 (+1) CT classification, the NRI increased to 0.975 and the IDI to 0.134. These findings indicate a significant improvement in predictive capability. However, clinical DCA indicated that the aforementioned combined models did not confer high-value benefits to patients. This may relate to the generally unfavorable three-month outcomes observed in patients with concomitant cerebral oedema included in this study. Further validation through multicenter, large-sample studies is warranted. Despite these limitations, considering that cerebral oedema-induced reduced cerebral perfusion and hypoxic brain tissue swelling exacerbate pathophysiological alterations (42), coupled with the urgent nature of RTAs in TBI patients, low examination compliance, and the current reality that many healthcare institutions cannot assess intracranial conditions via clear CT imaging, we contend that combining cerebral oedema parameters with the IMPACT model holds significant clinical value for evaluating the prognosis of TBI patients with RTAs. This approach may assist clinicians in early prognosis assessment, thereby guiding subsequent treatment decisions.

### Limitations

Naturally, this study has certain limitations. Firstly, owing to the constraints of a single-center study and small sample size, results may be subject to bias despite CT images being acquired using different equipment and parameters. Secondly, the study lacks external validation, necessitating future multi-center data for verification. Thirdly, due to the lack of clinical information in the samples, the effects of factors such as cerebral hypoxia/ischemia, cerebrovascular autoregulation impairment, and oxygen free radicals on the model were not discussed. Fourth, due to the unavailability of blood glucose and hemoglobin data in the current cohort, we failed to perform a comprehensive comparison among the IMPACT-core, IMPACT-extended, and IMPACT-lab model. Future prospective studies with complete laboratory data are warranted to conduct head-to-head comparisons of these three IMPACT-based predictive models. Fifth, although patients who underwent early decompressive craniectomy or hematoma evacuation were excluded, those receiving intracranial pressure probe placement and external ventricular drainage were not ruled out. These interventions may alter intracranial pressure dynamics, NCCT morphological features, and neurological functional outcomes, thereby introducing potential confounding effects on the observed correlations in this study. Finally, as a retrospective study, its assessment of long-term prognosis relies on clinical follow-up data, which may also introduce bias.

## Conclusion

In summary, the A5 (+1) CT classification outperformed the Marshall CT classification, Rotterdam CT score, and Helsinki CT score in assessing the three-month prognosis of TBI patients following RTAs, while demonstrating comparable performance to the IMPACT model. Furthermore, incorporating cerebral oedema parameters into the IMPACT model enhances predictive performance. External validation based on multicenter data is advocated and planned to demonstrate its generalizability.

## Data Availability

The raw data supporting the conclusions of this article will be made available by the authors, without undue reservation.
