# Supplementary material for: Prognostic value of oedema findings on head CT in traumatic brain injury following road traffic accidents
Source: Front Radiol. 2026 Oct 1;6:1941750. doi: 10.3389/fradi.2026.1941750 (PMC13671023; doi:10.3389/fradi.2026.1941750)
Supplement: Supplementary file 1 [file Table1.docx]

**Supplementary Materials**

**Supplementary** **Tables**

Table S1. Patients’ basic demographic and head AIS, stratified by the outcome

| Variable | Unfavorable outcome (n=84) | Favorable outcome (n=143) | *p* value |
| --- | --- | --- | --- |
| Gender, male, n (%) | 57 (67.9) | 109 (76.2) | 0.170 |
| Age, median (IQR) | 53 (21) | 47 (22) | 0.011 |
| Road user roles, n (%) |  |  | 0.056 |
| Motor vehicle driver | 9 (10.7) | 12 (8.4) |  |
| Passenger in motor vehicle | 6 (7.1) | 15 (10.5) |  |
| Motorcyclist | 27 (32.2) | 68 (47.5) |  |
| Pedestrian | 42 (50.0) | 48 (33.6) |  |
| GCS, n (%) |  |  | < 0.001 |
| 3-8 | 67 (79.8) | 15 (10.5) |  |
| 9-12 | 15 (17.8) | 32 (22.4) |  |
| 13-15 | 2 (2.4) | 96 (67.1) |  |
| Motor score, n (%) |  |  | < 0.001 |
| 1 | 11 (13.1) | 3 (2.1) |  |
| 2 | 25 (29.8) | 8 (5.6) |  |
| 3 | 28 (33.3) | 45 (31.4) |  |
| 4 | 18 (21.4) | 28 (19.6) |  |
| 5-6 | 2 (2.4) | 59 (41.3) |  |
| Pupillary light reflex, n (%) |  |  | < 0.001 |
| Normal | 43 (51.2) | 124 (86.7) |  |
| Unilateral disappearance | 12 (14.3) | 15 (10.5) |  |
| Bilateral disappearance | 29 (34.5) | 4 (2.8) |  |
| Head AIS, median (IQR) | 4 (1) | 3 (1) | < 0.001 |

IQR, interquartile range; GOS, Glasgow Coma Scale; AIS, Abbreviated Injury Scale.

Table S2. Patients’ CT imaging and NCCT scores, stratified by the outcome

| Variable | Unfavorable outcome (n=84) | Favorable outcome (n=143) | *p* value |
| --- | --- | --- | --- |
| Skull fracture, n (%) | 49 (58.3) | 89 (62.2) | 0.561 |
| Subdural hematoma, n (%) | 31 (36.9) | 38 (43.5) | 0.102 |
| Epidural hematoma, n (%) | 13 (15.5) | 20 (14.0) | 0.758 |
| ICH/Contusion, n (%) | 68 (80.9) | 60 (42.0) | < 0.001 |
| Numbers (IQR) | 1 (2) | 1 (1) | 0.010 |
| Numbers of cerebral lobes (IQR) | 2 (2) | 1 (1) | < 0.001 |
| Subarachnoid hemorrhage, n (%) | 55 (65.5) | 51 (35.7) | < 0.001 |
| Ventricular hemorrhage, n (%) | 14 (16.7) | 4 (2.8) | < 0.001 |
| Midline shift, n (%) | 29 (34.5) | 15 (10.5) | < 0.001 |
| Cerebral oedema, n (%) | 59 (70.2) | 49 (34.3) | < 0.001 |
| Marshall CT classification, n (%) |  |  | < 0.001 |
| Ⅰ | 0 (0) | 0 (0) |  |
| Ⅱ | 6 (7.2) | 42 (29.4) |  |
| Ⅲ | 20 (23.8) | 53 (37.0) |  |
| Ⅳ | 7 (8.3) | 5 (3.5) |  |
| Ⅴ + Ⅵ | 51 (60.7) | 43 (30.1) |  |
| Rotterdam CT score, n (%) | 3 (1) | 2 (1) | < 0.001 |
| Helsinki CT score, n (%) | 3 (3) | 2 (2) | < 0.001 |

ICH, intracerebral hemorrhage; IQR, interquartile range; NCCT, non-contrast computed tomography.

Table S3. Oedema parameters in patients with cerebral oedema, stratified by the outcome

| Variable | Unfavorable outcome (59) | Favorable outcome (49) | *p* value |
| --- | --- | --- | --- |
| Oedema volume, median (IQR) | 5.463 (11.677) | 1.343 (2.345) | < 0.001 |
| Oedema Index, median (IQR) | 2.864 (1.441) | 2.287 (0.692) | < 0.001 |
| A5 (+1) CT classification, n (%) |  |  | < 0.001 |
| 1 | 5 (8.5) | 19 (38.8) |  |
| 2 | 12 (20.3) | 19 (38.8) |  |
| 3 | 21 (35.6) | 7 (14.3) |  |
| 4-5 | 21 (35.6) | 4 (8.1) |  |

IQR, interquartile range.

Table S4. Predictive value of different prognostic models (all included patients)

| Control groups | Continuous NRI (95%CI) | *p* value | IDI | *p* value |
| --- | --- | --- | --- | --- |
| Model II vs Model I | 0.605 (0.358, 0.862) | < 0.001 | 0.027 (0.003, 0.051) | 0.026 |
| Model III vs Model I | 0.806 (0.557, 1.040) | < 0.001 | 0.099 (0.055, 0.140) | < 0.001 |
| Model IV vs Model I | 0.444 (0.169, 0.693) | < 0.001 | 0.064 (0.028, 0.096) | < 0.001 |
| Model V vs Model I | 0.868 (0.613, 1.105) | < 0.001 | 0.168 (0.111, 0.219) | < 0.001 |

Model Ⅰ (IMPACT): Age + Pupillary light reactivity + Motor score; Model Ⅱ: IMPACT + Marshall CT classification; Model Ⅲ: IMPACT + Rotterdam CT score; Model Ⅳ: IMPACT + Helsinki CT score; Model Ⅴ: IMPACT + A5 (+1) CT classification. IMPACT, International Mission for Prognosis and Analysis of Clinical Trials in TBI; NRI, net reclassification index; IDI, integrated discrimination improvement.

Table S5. Predictive value of different prognosis models (patients with concurrent cerebral oedema)

| Control groups | Continuous NRI (95%CI) | *p* value | IDI | *p* value |
| --- | --- | --- | --- | --- |
| Model II vs Model I | 0.349 (-0.215, 0.734) | 0.308 | 0.006 (-0.000, 0.055) | 0.516 |
| Model III vs Model I | 0.329 (-0.207, 0.888) | 0.030 | 0.040 (0.000, 0.138) | 0.058 |
| Model IV vs Model I | 0.425 (-0.031, 0.798) | 0.056 | 0.029 (-0.000, 0.131) | 0.072 |
| Model V vs Model I | 0.975 (0.540, 1.277) | < 0.001 | 0.134 (0.032, 0.286) | < 0.001 |
| Model VI vs Model I | 0.670 (0.250, 1.151) | 0.002 | 0.107 (0.033, 0.243) | < 0.001 |

Model Ⅰ (IMPACT): Age + Pupillary light reactivity + Motor score; Model Ⅱ: IMPACT + Marshall CT classification; Model Ⅲ: IMPACT + Rotterdam CT score; Model Ⅳ: IMPACT + Helsinki CT score; Model Ⅴ: IMPACT + A5 (+1) CT classification; Model Ⅵ: IMPACT + EI. IMPACT, International Mission for Prognosis and Analysis of Clinical Trials in TBI; EI, cerebral oedema; NRI, net reclassification index; IDI, integrated discrimination improvement.
